# Comparison of the Intestinal Microbiota During the Different Growth Stages of Red Swamp Crayfish (*Procambarus clarkii*)

**DOI:** 10.3389/fmicb.2021.696281

**Published:** 2021-09-13

**Authors:** Mengqi Xie, Shiyu Zhang, Lili Xu, Zhixin Wu, Junfa Yuan, Xiaoxuan Chen

**Affiliations:** ^1^Department of Aquatic Animal Medicine, College of Fisheries, Huazhong Agriculture University, Wuhan, China; ^2^Hubei Engineering Technology Research Center for Aquatic Animal Diseases Control and Prevention, Wuhan, China

**Keywords:** *Procambarus clarkii*, high-throughput sequencing, development stages, intestinal microbiota, histology

## Abstract

This study aimed to determine the effect of the growth stage of *Procambarus clarkii* on their intestinal microbiota. Intestinal samples of five different growth stages of *P. clarkii* (first instar, second instar, third instar, juvenile, and adult) from laboratory culture were analyzed through the Illumina MiSeq high-throughput sequencing platform to determine the intestinal microbiome of crayfish. The alpha diversity decreased along with the growth of the crayfish, with the relative abundance of the microbiota changing among stages; crayfish at closer development stages had a more comparable intestinal microbiota composition. A comparative analysis by principal component analysis and principal coordinate analysis showed that there were significant differences in the intestinal microbiota of crayfish among the different growth stages, except for the first two stages of larval crayfish, and the intestinal microbiota showed a consistent progression pattern from the larval stage to the juvenile stage. Some microbiota showed stage specificity, which might be the characteristic microbiota of different stages of growth. According to FAPROTAX functional clustering analysis, the three stages of larvae were clustered together, while the juvenile and adult stages were clustered separately according to the growth stage, indicating that, in the early stages of larval development, the function of the intestinal flora was similar; as the body grew and developed, the composition and function of the intestinal microbiota also changed.

## Introduction

The main digestive organ of animals is the intestine, where a rich and complex microbial community colonizes. Homeostasis between the intestinal microbiota and the host has built up through long-term evolution (Bates et al., 2006), and much evidence indicates that the intestinal microbiota plays an essential role in helping the host maintain normal physiological processes (Round and Mazmanian, 2009; Bajaj et al., 2018). In fact, many intestinal microbial species participate in the digestion of food, such as starch and cellulose, by secreting digestive enzymes to provide short-chain fatty acids, amino acids, and vitamins for the host (Bäckhed et al., 2004, 2007; Sonnenburg et al., 2005; LeBlanc et al., 2012). The normal colonization of the host intestine with commensal microbes has been proven to drive the development of the immune system (Cebra, 1999). Some intestinal microbiomes also affect the processes of immunoglobulin secretion, non-specific immunity, and immune response to maintain the normal function of the immune system (Rosshart et al., 2017).

There are many factors that affect the intestinal microbiota, such as the host genotype, stage of host development, host diet, and environmental microbiota (LeaMaster, 1997; Mouchet et al., 2011; Yan et al., 2012; Rungrassamee et al., 2013; Miyake et al., 2014; Bakke et al., 2015). Additionally, the structure of the intestine changes as the host grows (Zhang and Li, 2018), and the composition of the intestine microbiota also varies over time (Amato et al., 2017). As reported in humans, the composition, abundance, and distribution of gut microbiota change with increasing age (Mariat et al., 2009; Štšepetova (Shchepetova) et al., 2011). Moreover, the intestinal microbiota is relatively stable in adults, and its structure changes more obviously in old people (Claesson et al., 2011). In other mammals, such as cattle and sheep, significant differences in the digestive tract microbiota among different stages of growth have been reported (Pang et al., 2018). Therefore, studying the factors and patterns that affect intestinal microbes is of great significance for regulating the interactions between the host organism and the intestinal microbiota.

Most aquatic animals reproduce through oviposition, which makes the intestinal ecosystem of these animals quite unique. Initially, they are separated from the environment by the chorionic membrane, and the intestine opens to the water and is exposed to the environmental microbiota within a few days after hatching (Castaneda et al., 2006). In the early development of aquatic animals (*e*.*g*., at the larval stage), the colonization of bacteria is complicated, and the intestinal microbiota shows different structural characteristics throughout the various stages of development (Kohl et al., 2013; Wong et al., 2015; Jiménez and Sommer, 2017). Usually, studies of intestinal microbiota in aquatic animals have focused on fish. As reported previously, the composition of intestinal microbiota in many fish species is affected by their development stage and diet (Savaş et al., 2005; Bakke et al., 2015; Stephens et al., 2015; Wong et al., 2015; Bledsoe et al., 2016; Parris et al., 2016; Yan et al., 2016). Unlike chordates, crustaceans molt many times throughout their life. Moreover, many of them have morphological and dietary changes at the early stage of development, which induce a quite complex effect on the intestinal microbiota – for example, the Chinese mitten crab (*Eriocheir sinensis*) has been reported to have significantly different core microbiota at different stages of development, and the diversity of the gut microbiota is the highest in juvenile crabs and decreases with age (Wang et al., 2018).

Since entering China in the 1930s, the red swamp crayfish (*Procambarus clarkii*), a kind of freshwater crayfish, has gradually become one of the most important aquatic products in China. *P. clarkii* has a complex early life history due to changes in diet and morphology caused by molting. By the time of hatching, most organs have been specified, except for the uropod and the first pleopod; the first-instar larvae rely on the yolk for nutrition. After the first molting, the uropods of the second-instar larvae appear, and the larvae begin ingesting food. After the second molting, the first pleopods of the third-instar larvae appear. At this stage, the larvae have fully developed, and exogenous food is ingested as the only source of nutrition since the yolk has been depleted (Huner, 1988; Guo and Zhu, 1997; Castaneda et al., 2006; Chen, 2015). In recent years, *P. clarkii* has been in short supply, and consumers have higher requirements regarding their production and quality. As an important factor impacting the health of the host, it is still unclear how the intestinal microbiota changes and what the effect is at different stages of *P. clarkii* growth. In order to obtain important basic data and provide guidance and basis for searching for factors and patterns related to changes in the intestinal microbiota in *P. clarkii*, this study aimed to analyze the composition of the intestinal microbiota and the histology of the intestine in *P. clarkii* at different development stages and to identify some stage-specific microbiota.

## Materials and Methods

### Crayfish Collection

Red swamp crayfish were obtained from a crayfish culture base (30.39° N, 113.77° E, Wuhan, China) and brought to the laboratory alive. Groups of 10 crayfish were cultured in a sterile tank (70 cm × 45 cm × 17 cm, length × width × height). The ratio of males to females was set at 1:1. During the experiment, the crayfish were fed with commercial pellet feed (Tongwei Group, Wuhan). The culture water was kept at 25 ± 1°C and a depth of 5 ± 1 cm with a 12 h dark/light cycle. The experiment ran for 8 weeks to include crayfish breeding and egg hatching. The development stages of larvae were observed under an optical microscope.

### Histology

According to a previous report (Guo and Zhu, 1997), the samples covered five key development stages: first-instar (FI) larvae, second-instar (SI) larvae, third-instar (TI) larvae, juvenile (J), and adult (A). For histological analysis, a section of the intestine was cut from an adult crayfish, and the entire body was sampled in larval and juvenile crayfish due to their small size. Next, the segments were immersed in fixative (4% paraformaldehyde) for 2 h. The specimens were dehydrated with a graded series of ethanol, infiltrated and embedded with paraffin, and then sectioned. The sections were stained by hematoxylin–eosin staining.

### Sampling of Intestines and DNA Extraction

A total of 120 larvae (40 larvae at each instar), 20 juveniles, and 12 adults were sampled randomly. The body surface of the red swamp crayfish was washed and disinfected using 75% ethanol. Then, the intact hindguts were isolated with sterile forceps and scissors under aseptic operation conditions. The total genomic DNA was isolated from the samples according to the instructions of the Fecal DNA Extraction Kit (Aidlab Biotech, Beijing, China). Some of the gut tissues derived from the same development stage (no significant differences in individual size) were pooled for DNA extraction due to the low tissue mass.

### Polymerase Chain Reaction Amplification

The V3/V4 region of the 16S rRNA gene was amplified with the primers 335F and 769R (335F, 5′-CADACTCCTACGGGAGGC-3′; 769R, 5′-ATCCTGTTTGMTMCCCVCRC-3′). The PCR conditions were denaturation at 95°C for 5 min, followed by 35 cycles consisting of 95°C for 30 s, 50°C for 30 s, and 72°C for 40 s, and a final elongation step at 72°C for 7 min. The amplification products were analyzed by electrophoresis in 1.8% agarose gels. Then, the DNA in each band was excised and purified using the To Pure^TM^ Gel Extraction Kit (Gene Tech, Shanghai, China). Each purified PCR product was subjected to Illumina-based high-throughput sequencing (BGI, Shenzhen, China). The sequencing platform and instrument used was Illumina HiSeq 2500.

### Bioinformatic Analysis

To obtain more accurate and reliable results in the subsequent bioinformatic analysis (Fadrosh et al., 2014), the data were preprocessed. According to the overlap between the paired-end reads, the paired-end sequencing data obtained by HiSeq sequencing were merged into raw tags using FLASH v1.2.7. Next, the clean tags were filtered from the raw tags using Trimmomatic v0.33. Sequencing errors and chimeras were detected and removed, and the reads that could not be assembled were discarded. The clean tags were clustered into operational taxonomic units (OTUs) by USEARCH (v10.0) at 97% similarity levels (Edgar, 2013), and the OTU sequences were searched against the Ribosomal Database Project (RDP) using the RDP classifier (v2.2) (R.Cole’s et al., 2013) with a confidence threshold of 80%. Rarefaction curves were plotted for each sample to determine the abundance of communities and sequencing data of each sample. The alpha diversity index was determined using Chao1 (total species richness), abundance-based coverage estimator (ACE), the Shannon index, and the Simpson index in Mothur (v1.31.2) (Schloss et al., 2009). A community bar plot analysis conducted on the BMKCloud platform was carried out to show the relative abundance of the gut bacterial community among the samples at the phylum and genus levels. Differences between groups were analyzed by one-way analysis of variance, followed by Duncan multiple-comparison tests using SPSS v26.0; *p* < 0.05 was considered statistically significant. The results were presented as mean ± standard deviation. Beta diversity measurements including principal component analysis (PCA) and principal coordinate analysis (PCoA) based on the Bray–Curtis distance were calculated. Microbiota functions were predicted through functional annotation of prokaryotic taxa (FAPROTAX).

## Results

### Sequencing Depth and Alpha Diversities

**FIGURE 1:**
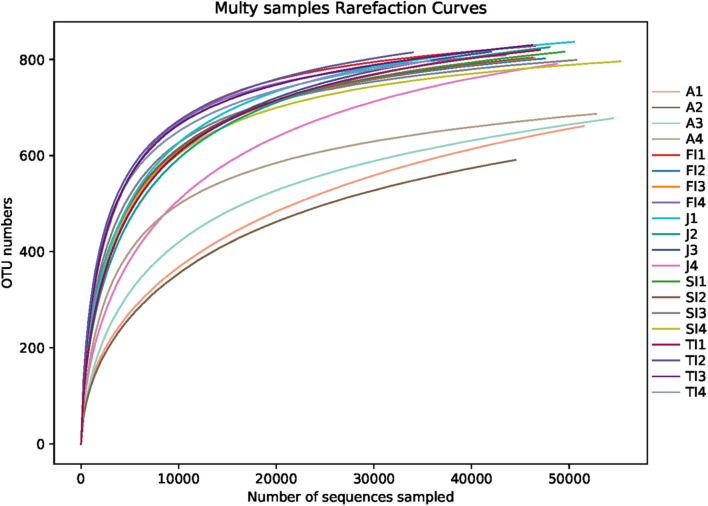
Rarefaction curves of samples. FI, first instar larvae; SI, second instar larvae; TI, third instar larvae; J, juvenile; A, adult.

After removing the low-quality reads, a total of 1,400,062 reads were obtained from 20 samples. The average sequence length was 403 bp. The number of reads for the various samples at different stages ranged from 74,784 to 78,735, of which 60,696 to 75,933 reads remained after rarefaction. A total of 956 OTUs were obtained, ranging from 591 to 837, in the crayfish intestinal samples. The Good’s coverages of all samples were greater than 99%, suggesting that the sequencing depth of all samples was sufficient to represent the bacterial community in the crayfish intestine. The rarefaction curves also showed that a sufficient sampling depth was achieved (Figure 1).

**FIGURE 2:**
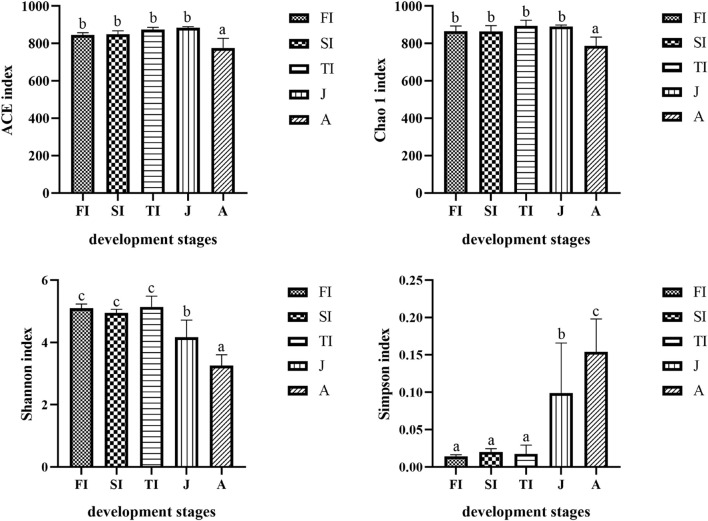
Comparison and analysis of bacterial composition richness (Chao 1 index and ACE index) and diversity (Shannon index and Simpson index). The alpha diversity values were compared in the groups of different development stages. In every single histogram, column values with no letter or the same letters mean no significant difference between them (*P* > 0.05), while with different small letters mean significant difference (*P* > 0.05).

The Chao1 and ACE indexes of the adult samples were lower than those of the other samples (*p* < 0.05), whereas there were no significant differences between the juvenile and larval samples (*p* > 0.05). Moreover, the Shannon indexes of the adult samples were lower than those of the juvenile samples, and those of the juvenile samples were lower than those of the larval samples (*p* < 0.05); however, there were no significant differences among the different stages of larval samples (*p* > 0.05). The Simpson indexes of the adult samples were higher than those of the juvenile samples, and those of the juvenile samples were higher than those of the larval samples (*p* < 0.05), but there were no significant differences among the different stages of larval samples (*p* > 0.05) (Figure 2).

### Microbial Community Compositions and Dominant Bacterial Taxa

**FIGURE 3:**
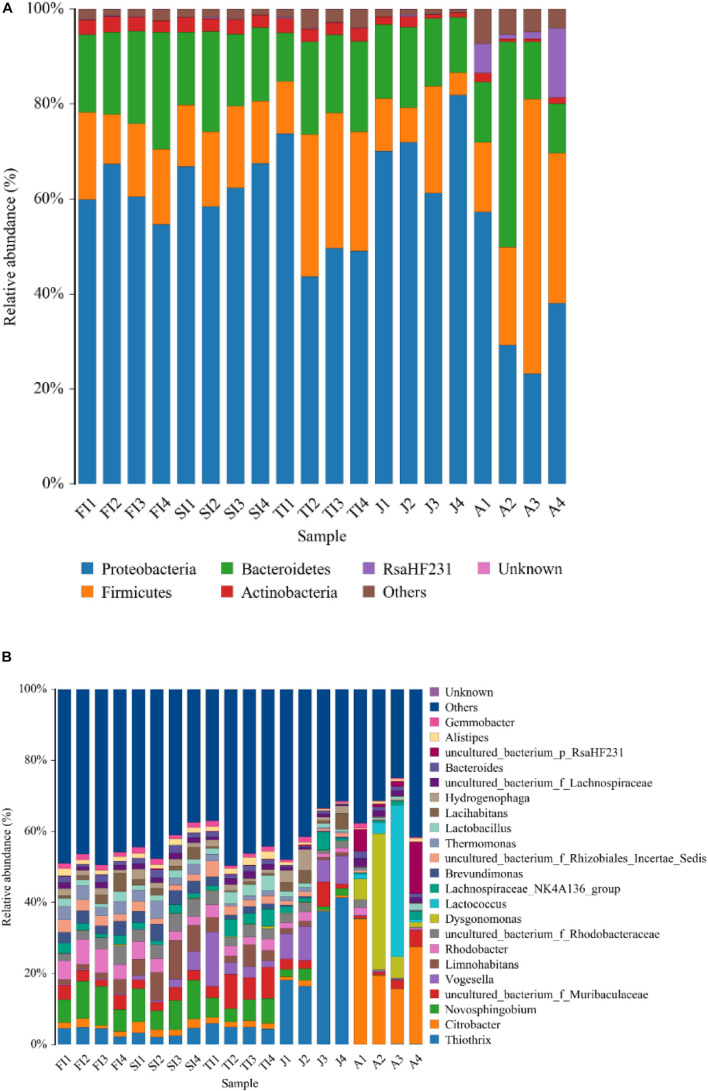
Community bar plot analysis showing the relative abundance of gut bacterial community among the samples by Mothur at the phylum **(A)** level and genus **(B)** level. Less than 1% abundance of the phyla/genera was merged into “others.”

At the phylum level, the intestinal bacteria of the FI and SI groups were dominated by Proteobacteria, which accounted for more than 60%. Other dominant phyla included Bacteroidetes, Firmicutes, Actinobacteria, and Fusobacteria. The dominant bacteria of the TI group were similar to those of the FI and SI groups. However, the abundance of Fusobacteria was greater at the FI stage. At the juvenile stage, the abundance of Proteobacteria increased significantly (*p* < 0.05), the abundance of Actinobacteria and Fusobacteria decreased significantly (*p* < 0.05), and the abundance of Fusobacteria dropped below 1%. At the adult stage, Proteobacteria was still the most dominant phylum, but its abundance decreased significantly (*p* < 0.05), and the abundance of many other phyla, like Tenericutes, increased and became dominant (Figure 3A and Table 1A).

**TABLE 1A:** The relative abundance of gut abundant microbiota among the samples at the phylum level (A) and genus level (B).

FI		SI		TI		J		A	
Phylum	Precent	Phylum	Precent	Phylum	Precent	Phylum	Precent	Phylum	Precent
Proteobacteria	60.74	Proteobacteria	63.97	Proteobacteria	55.25	Proteobacteria	71.64	Proteobacteria	37.04
Bacteroidetes	19.36	Bacteroidetes	16.64	Firmicutes	23.00	Bacteroidetes	14.60	Firmicutes	31.94
Firmicutes	14.98	Firmicutes	14.73	Bacteroidetes	15.87	Firmicutes	11.04	Bacteroidetes	18.60
Actinobacteria	2.94	Actinobacteria	2.87	Actinobacteria	2.78	Actinobacteria	1.47	RsaHF231	5.95
Fusobacteria	1.00			Fusobacteria	1.38			Tenericutes	2.30
								Epsilonbacteraeota	1.42
								Actinobacteria	1.13

At the genus level, the dominant microbiota of the FI and SI groups included *Novosphingobium*, *Rhodobacter*, *Thiothrix*, *Thermomonas*, an unclassified genus of Rhodobacteraceae, and *Brevundimonas*. The abundance of *Vogesella* increased significantly at the TI stage (*p* < 0.05), and the abundance of *Hydrogenophaga* increased significantly at the juvenile stage (*p* < 0.05). At the adult stage, *Citrobacter* (24.42%) became the most dominant genus, and some other genera, like *Lactococcus*, increased significantly (12.70%) (*p* < 0.05) (Figure 3B and Table 1B).

**TABLE 1B:** The relative abundance of gut abundant microbiota among the samples at the phylum level (a) and genus level (b).

FI		SI		TI		J		A	
Genus	Precent	Genus	Precent	Genus	Precent	Genus	Precent	Genus	Precent
*Novosphingobium*	8.56	*Novosphingobium*	8.55	*f*_*Muribaculaceae*	6.65	*Thiothrix*	27.98	*Citrobacter*	24.42
*Rhodobacter*	5.83	*Limnohabitans*	7.73	*Vogesella*	6.35	*Vogesella*	7.73	*Lactococcus*	12.70
*Thiothrix*	4.04	*f*_Rhodobacteraceae	4.34	*Novosphingobium*	5.59	*f*_*Muribaculaceae*	3.21	*Dysgonomonas*	11.81
*Thermomonas*	3.43	*Brevundimonas*	3.79	*Thiothrix*	5.09	*Hydrogenophaga*	3.14	*p*_RsaHF231	5.95
*f*_*Muribaculaceae*	3.28	*Rhodobacter*	3.41	*Limnohabitans*	3.97	*Lacihabitans*	2.94	*f*_*Muribaculaceae*	2.13
*f*_*Rhodobacteraceae*	3.20	*Thermomonas*	3.21	*f*_*Rhodobacteraceae*	3.29	*Novosphingobium*	2.16	*f*_*Lachnospiraceae*	1.73
*Brevundimonas*	3.14	*Thiothrix*	3.18	*Lachnospiraceae_NK4A136*	3.24	*f*_*Rhodobacteraceae*	2.02	*Bacteroides*	1.34
*f*_*Rhizobiales_Incertae_Sedis*	2.91	*f*_*Muribaculaceae*	2.86	*Lactobacillus*	2.98	*Lachnospiraceae_NK4A136*	1.95	*Lachnospiraceae_NK4A136*	1.12
*Lacihabitans*	2.38	*Vogesella*	2.43	*Rhodobacter*	2.94	*Rhodobacter*	1.66		
*Lachnospiraceae_NK4A136*	2.05	*Citrobacter*	2.38	*f*_*Rhizobiales_Incertae_Sedis*	2.80	*Limnohabitans*	1.21		
*Limnohabitans*	2.05	*Lacihabitans*	2.08	*Brevundimonas*	1.88	*Lactobacillus*	1.08		
*Lactobacillus*	1.88	*f*_*Rhizobiales_Incertae_Sedis*	1.96	*Alistipes*	1.82	*f*_*Rhizobiales_Incertae_Sedis*	1.07		
*Citrobacter*	1.63	*Hydrogenophaga*	1.91	*Citrobacter*	1.64				
*Hydrogenophaga*	1.58	*Bacteroides*	1.81	*f*_*Lachnospiraceae*	1.51				
*Alistipes*	1.45	*Lachnospiraceae_NK4A136*	1.61	*Bacteroides*	1.44				
*Gemmobacter*	1.44	*Lactobacillus*	1.60	*Thermomonas*	1.34				
*Bacteroides*	1.43	*f*_*Lachnospiraceae*	1.53	*Hydrogenophaga*	1.31				
*f*_*Lachnospiraceae*	1.39	*Gemmobacter*	1.48	*Gemmobacter*	1.22				
		*Alistipes*	1.42						

**FIGURE 4:**
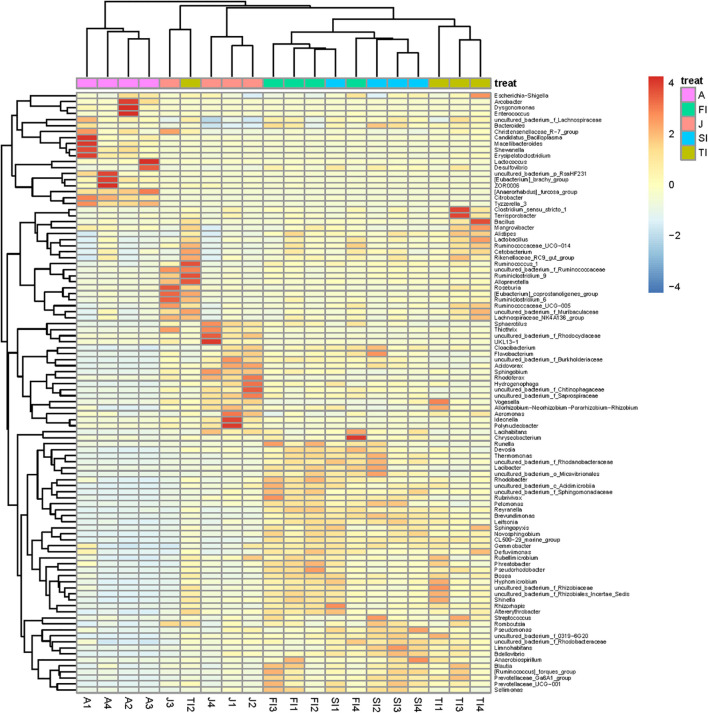
Heatmap analysis of the bacterial species in all samples. The color of the bar represents the relative abundance of each bacterial genus in all samples. The longitudinal clustering indicates the similarity of all species among different samples.

To determine the similarities and differences of the intestinal community compositions during the different development stages, we performed hierarchical clustering analysis according to the relative abundance of each species at the genus level (Figure 4). The crayfish at closer development stages had comparable intestinal microbiota compositions. The FI group and the SI group were clustered together, and their intestinal bacteria were both dominated by Proteobacteria, including *Novosphingobium*, *Limnohabitans*, an unclassified genus of Rhodobacteraceae, *Brevundimonas*, *Rhodobacter*, *Thermomonas*, and *Thiothrix*. The adult group had the lowest similarity compared to the other groups, and many bacteria not dominant at previous stages became dominant at this stage. In addition, some bacteria dominant at the larval stages greatly decreased, like *Thermomonas*, *Novosphingobium*, *Rhodobacter*, *etc*.

### Comparative Analysis Among the Different Development Stages

**FIGURE 5:**
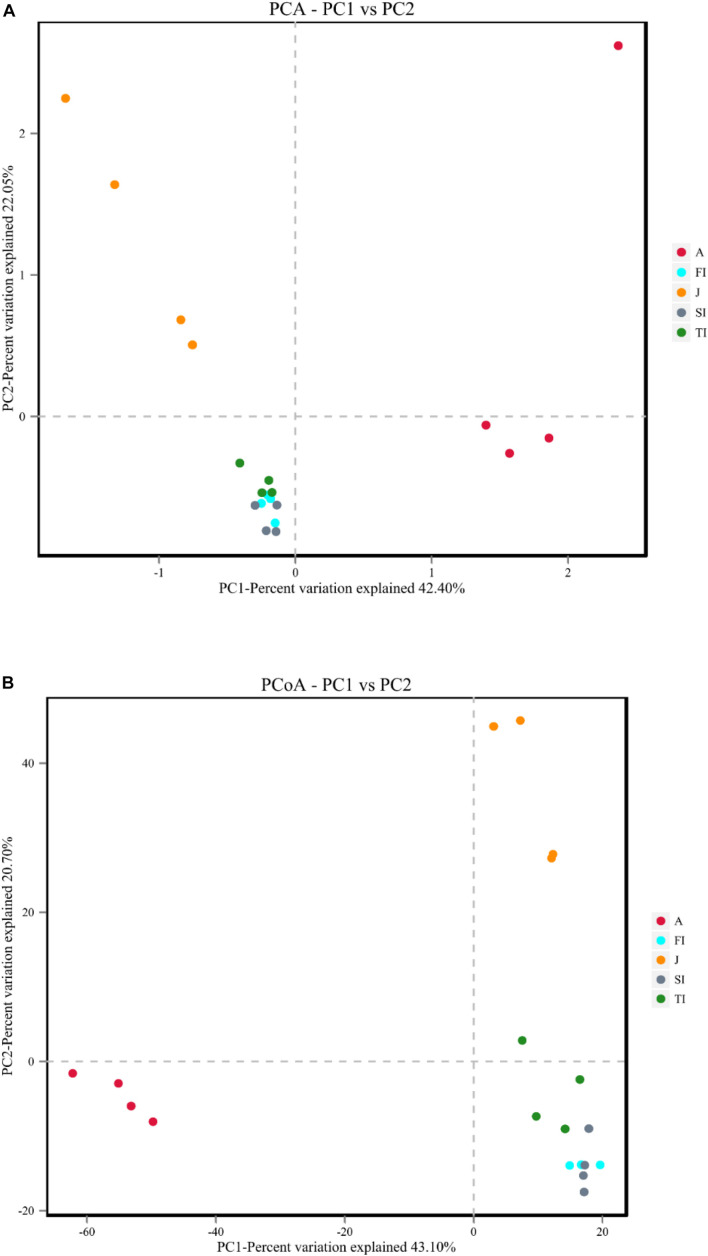
**(A)** Principal component analysis plot of intestinal microbiota at different development stages. **(B)** Principal coordinate analysis plots of Bray–Curtis comparing the intestinal microbiota at different development stages.

The differences among samples were reflected by analyzing the OTU composition of the different samples with PCA and PCoA (Figure 5). PCA showed that the intestinal microbiota at the larval stages were clustered together. The larval, juvenile, and adult stages were clustered separately according to the development stage, and the juvenile and adult groups were discriminated by the pc1 axis (42.40%) (Figure 5A). PCoA also showed the grouping of crayfish intestinal microbiota within each development stage, yet with much overlap across FI and SI. At the larval and juvenile stages, the intestinal microbiota showed a consistent progression trend: they had similar coordinates on the pc1 axis (43.10%) and were discriminated by the pc2 axis (20.70%) (Figure 5B). The distinct intestinal microbiota among the development stages were confirmed by the analysis of similarities based on the Bray–Curtis distance matrix, and the results demonstrated that there were significant differences among the various development stages (*p* < 0.05), except between FI and SI (Table 2).

**TABLE 2:** Analysis of similarities based on Bray–Curtis distance matrix.

	FI	SI	TI	J	A
FI		*R* = 0.53, *P* = 0.06	*R* = 0.69, *P* = 0.03	*R* = 1.00, *P* = 0.03	*R* = 1.00, *P* = 0.03
SI	*R* = 0.53, *P* = 0.06		*R* = 0.66, *P* = 0.03	*R* = 0.99, *P* = 0.03	*R* = 1.00, *P* = 0.03
TI	*R* = 0.69, *P* = 0.03	*R* = 0.66, *P* = 0.03		*R* = 0.95, *P* = 0.03	*R* = 1.00, *P* = 0.02
J	*R* = 1.00, *P* = 0.03	*R* = 0.99, *P* = 0.03	*R* = 0.95, *P* = 0.03		*R* = 1.00, *P* = 0.04
A	*R* = 1.00, *P* = 0.03	*R* = 1.00, *P* = 0.03	*R* = 1.00, *P* = 0.02	*R* = 1.00, *P* = 0.04	

*The closer the R value is to 1, the higher is the difference between groups than that within groups; the smaller the R value is, the less significant is the difference between them. P value less than 0.05 indicates high reliability of the test.*

**FIGURE 6:**
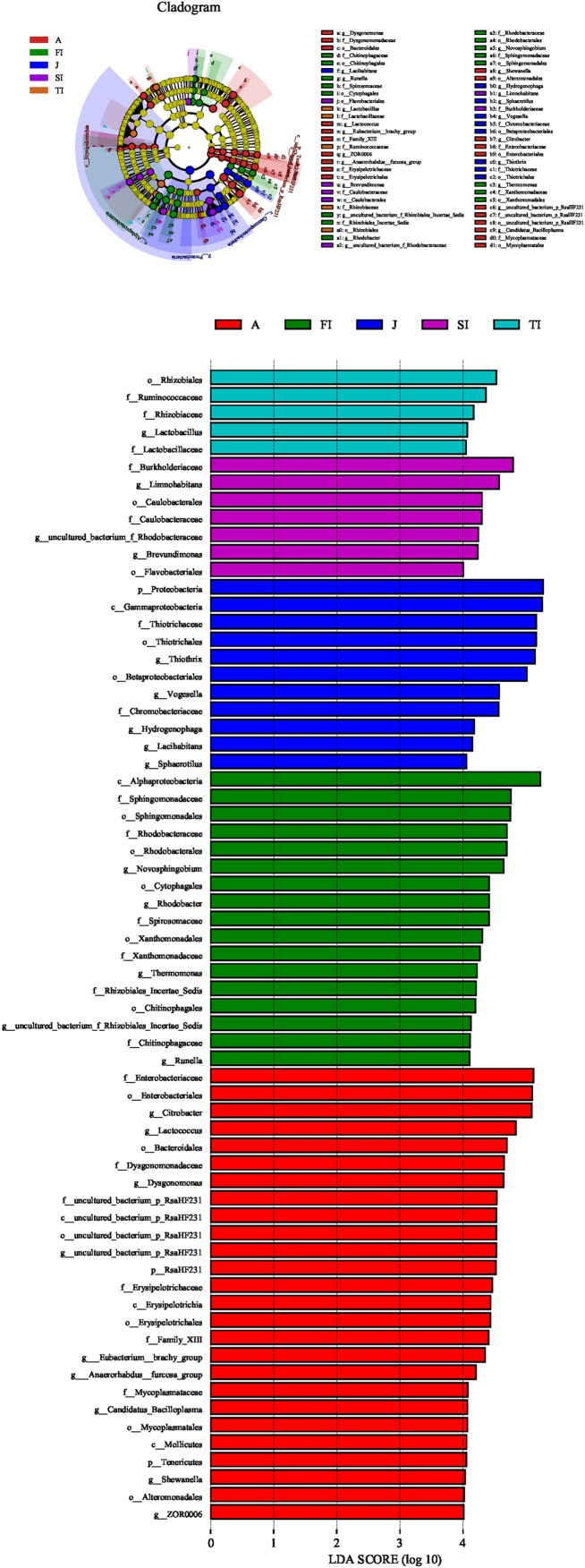
Linear discriminant analysis taxonomic cladogram showing the discriminatory taxa at each development stage of crayfish. The figure shows the taxa whose linear discriminant analysis score was higher than 4.0. The discriminatory taxon nodes were colored, and the branch areas were shaded according to the highest-ranked group for that taxon. If the taxon was not significantly differentially represented among sample groups, the corresponding node was colored yellow.

Based on the above-mentioned analysis, we used the linear discriminant analysis effect size to determine the potential discriminating taxon between the different development stages (Figure 6). A total of 66 bacterial groups were found to have significant differences among the various development stages. Some bacterial groups exhibited stage-specific signatures. *Lactococcus* remained stable from the FI stage to the adult stage (*p* > 0.05). *Rhodobacter* decreased sharply from the FI stage to the adult stage (*p* < 0.05). *Thermomonas* and *Novosphingobium* decreased gradually from the FI stage to the adult stage. *Limnohabitans*, *Brevundimonas*, and an unclassified genus of Rhodobacteraceae had the highest level at the SI stage and then decreased gradually along with development. An unclassified genus of Muribaculaceae was at a high level only at the TI stage, but it was at low levels at the other stages (*p* < 0.05). *Thiothrix* was at a low level at the larval and adult stages but at a high level at the juvenile stage (*p* < 0.05). *Vogesella* increased gradually from the FI stage to the juvenile stage and reached the highest level at the juvenile stage but sharply decreased at the adult stage (*p* < 0.05). *Dysgonomonas*, *Citrobacter*, and an unclassified genus of RsaHF231 all had a low abundance at the first four stages, but they sharply increased at the adult stage (*p* < 0.05).

### Microbiota Functional Prediction Analysis

**FIGURE 7:**
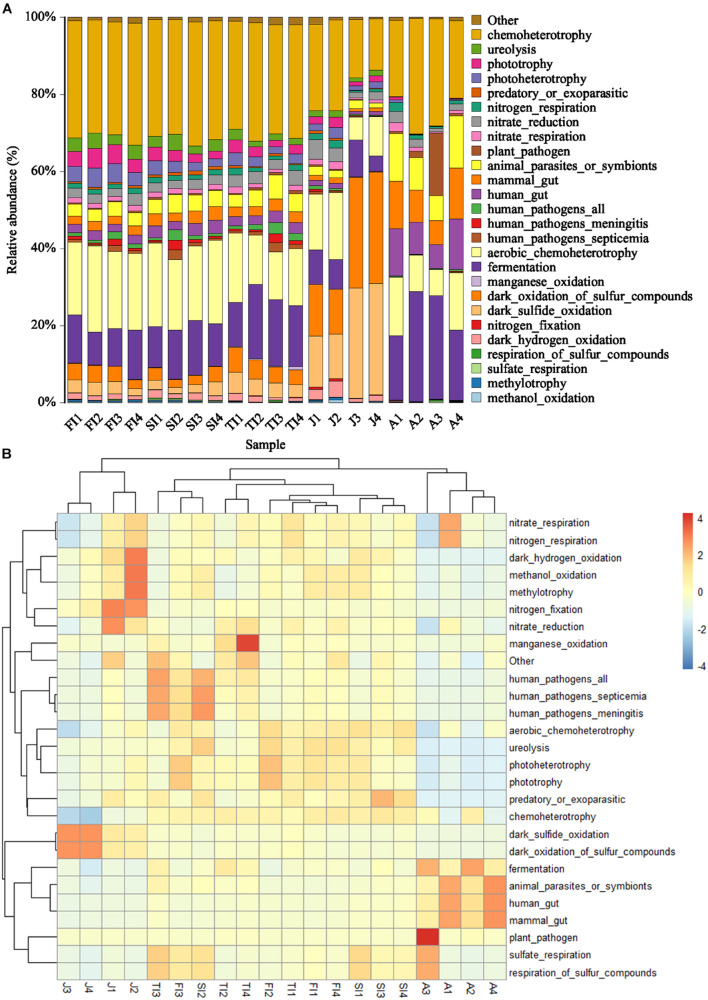
Histogram **(A)** and Heatmap **(B)** showing the predicted function potentials of crayfish intestinal microbiota across development stages through FAPROTAX prediction.

The similar microbiota functional profiles were predicted through FAPROTAX (Figure 7). At the three larval stages, the most dominant functions of the intestinal microbiota were mainly chemoheterotrophy (30.02 ± 0.75%), aerobic chemoheterotrophy (18.35 ± 3.85%), and fermentation (12.99 ± 2.97%), with a total relative abundance of more than 60%, while the relative abundances of the other functions were all less than 5%. The dominant functional profiles of juveniles included dark oxidation of sulfur compounds (20.69%), dark sulfide oxidation (20.56%), chemoheterotrophy (18.55%), aerobic chemoheterotrophy (11.97%), *etc*. Meanwhile, the dominant functional profiles of adults included chemoheterotrophy (24.39%), fermentation (22.58%), aerobic chemoheterotrophy (11.56%), animal parasites or symbionts (10.19%), *etc*. In functional clustering, the three larval stages were clustered together, while the juvenile and adult stages were clustered separately according to the growth stage.

### Histology of the Intestine

As time passed, the intestine of *P. clarkii* developed (Supplementary Figure 1). At the FI stage, the intestinal tissue was incomplete, with most structures not yet developed, except for a small number of epithelial cells (Supplementary Figure 1A). At the SI stage, the intestine thickened, the lumen expanded, the epithelial cells increased, and a circular layer of striated muscles appeared (Supplementary Figure 1B). At the TI stage, connective tissue and a small number of longitudinal layers of striated muscle appeared (Supplementary Figure 1C). At the juvenile stage, longitudinal layers of striated muscle appeared in large numbers, and the intestinal structure was present; however, the intestinal lumen was still hollow, and the longitudinal ridges were not closely arranged (Supplementary Figure 1D). At the adult stage, all of the structures of the intestine were fully developed, and the longitudinal ridges were arranged closely, filling the entire intestine (Supplementary Figures 1E,F).

## Discussion

The intestinal microbiota changes throughout the life cycle and is affected by many factors, including genetic background, physiological state, and biological habits like the age of the host, health, food intake, and living environment (Ni et al., 2012; Xiong et al., 2015, 2017). Although the intestinal microbiota has been proven to play a crucial role in the immunity and metabolism of the host, it is still unclear how it changes and what its effect is at different stages of host growth.

For most mammals, the intestinal microbiota tends to be rich and stable with host development (Palmer Davis et al., 2007; Wang et al., 2019). Nevertheless, due to their living environment, oviparous, aquatic animals have more differences in their intestinal microbiota among the various development stages than terrestrial animals (Wang et al., 2018). For aquatic animals like *Ctenopharyngodon idellus*, *Siniperca chuatsi*, and *Silurus meridionalis*, the intestinal microbiota diversity declines with host development (Stephens et al., 2015; Wong et al., 2015; Yan et al., 2016). Furthermore, some studies have demonstrated that the bacterial alpha diversity indexes show a U-shaped pattern along with host development in *Litopenaeus vannamei*, that is, the diversity of the intestinal microbiota reveals a downward trend in the early developmental period (Wang et al., 2020). *P. clarkii* has a very complex early life history, with changes in feeding habits and morphology, as well as later physiological states such as sexual maturation that may have an impact on the intestinal microbiota. In this study, the intestinal microbiota was abundant at the larval stages, and the alpha diversity decreased along with the development stages. The microbiota of eggs might be important for the establishment of the larval indigenous microbiota, and the egg and larval surfaces provide a suitable micro-environment for bacterial growth, which might be responsible for the association of greater numbers of bacteria with larvae (Stevenson, 1977; Hansen and Olafsen, 1989; Hameed, 1993). The highly diverse microbiota in larvae might also be related to the unstable and susceptible larval intestinal microbiota. After beginning to ingest food, the larvae feed on plankton (Huner, 1988; Guo and Zhu, 1997; Castaneda et al., 2006; Chen, 2015), and the bacteria will enter the intestine along with various foods, thus making the intestinal microbiota more complicated. At the adult stage, with the physiological state fully developed, a less diverse microbiota is needed for metabolism and immune function (Yan et al., 2016). The intestinal microbiota of the adult crayfish was relatively stable and had the ability to resist colonization of passing bacteria, which might also lead to the limited diversity of the intestinal microbiota in adult crayfish (Carassou et al., 2012; Zheng et al., 2017).

The FI and SI larvae had a similar intestinal microbiota composition, both of which were dominated by Proteobacteria, including *Novosphingobium*, *Limnohabitans*, an unclassified genus of Rhodobacteraceae, *Brevundimonas*, *Rhodobacter*, *Thermomonas*, and *Thiothrix*. At these stages, due to the presence of the egg yolk, the larvae mainly absorbed endogenous nutrition, and their gut bacteria were mainly derived from environmental microorganisms (Rungrassamee et al., 2014). The dominant bacteria are usually found in the water and are important microbiota at the larval stages. During the TI period, when the yolk degenerates and the crayfish shifts to ingest exogenous nutrition and begins to feed on plankton, the intestinal flora was impacted by food intake and differed from the first two stages; an unclassified genus of Muribaculaceae (6.65%), *Vogesella* (6.35%), *Novosphingobium* (5.59%), *Thiothrix* (5.09%), *Limnohabitans* (3.97%), an unclassified genus of Rhodobacteraceae (3.29%), Lachnospiraceae_NK4A136 (3.24%), *etc*. were dominant during the TI stage. In addition to the previously existing dominant flora, the relative abundance of bacteria such as *Vogesella* increased and became dominant in the microbiota. During the juvenile stage, the crayfish started to ingest pelleted feed, and the intestinal microbiota differed significantly from the previous ones, being dominated by *Thiothrix* (27.98%), *Vogesella* (7.73%), an unclassified genus of Muribaculaceae (3.21%), and *Hydrogenophaga* (3.14%) in the juvenile period. During the adult stage, with sexual maturity, the intestinal microbiota changed dramatically along with the altered physiological status, and some bacteria that were not dominant in the larvae became dominant, such as *Citrobacter* (24.42%), *Lactococcus* (12.7%), *Dysgonomonas* (11.81%), an unclassified genus of RsaHF231 (5.95%), *etc*.

Throughout the growth cycle of crayfish, some microbiota showed stage-specific signatures – for example, *Lactococcus* remained stable from the FI stage to the adult stage (*p* > 0.05) as a conserved flora in the intestine of *P. clarkii*. Many studies have shown the potential effects of *Lactococcus* on the growth and digestive enzyme activities of shrimp (Adel et al., 2017). In addition, *Lactococcus* belonging to Firmicutes participates in the degradation of undigested carbohydrates and amino acids into short-chain fatty acids, promoting the metabolic function of crayfish (Lopetuso et al., 2013; Wu et al., 2021) and thus playing an important role throughout the growth stages of *P. clarkii*.

*Rhodobacter* decreased sharply from the FI stage to the adult stage (*p* < 0.05). *Rhodobacter*, belonging to the Rhodobacteraceae family, is commonly found in the gut of healthy crustaceans. Rhodobacteraceae also decreased sharply from the FI stage to the adult stage in this study (*p* < 0.05). Rhodobacteraceae have been reported to be usually dominant in the intestinal flora in shrimps after initial feeding, and most of them have a *de novo* pathway for vitamin B12 synthesis, which has been shown to be essential for the shrimp diet (Yao et al., 2018). Furthermore, Rhodobacteraceae have the potential to promote the biosynthesis and metabolism of a wide range of organic matter and may be involved in the metabolism of organic matter in the digestive tract of larvae (Zheng et al., 2017). The decline of *Rhodobacter* and Rhodobacteraceae throughout growth suggested the greater possibility of their colonization in the intestine of crayfish larvae, and *Rhodobacter* and Rhodobacteraceae might be the characteristic microbiota of the larval stage.

*Thermomonas* and *Novosphingobium* decreased gradually from the FI stage to the adult stage. These two genera are usually found in natural water. *Thermomonas*, a heterotrophic denitrifying bacterium, is abundant in the aquatic environment and sediment (Li et al., 2020). *Novosphingobium* produces prolyl endopeptidase, which is an enzyme that degrades lignin produced by plants and is the major component of terrigenous organic carbon discharged into aqueous environments (Gass et al., 2007; Ohta et al., 2015). *Novosphingobium* also has been reported to be the dominant flora of the intestinal microbiota in *Penaeus vannamei*, especially in larvae and juvenile *Penaeus monodon* (Rungrassamee et al., 2014; Panigrahi et al., 2019). Its potential for colonization in shrimp and crayfish, with a bias toward the larval intestine, warrants further study.

*Limnohabitans*, *Brevundimonas*, and an unclassified genus of Rhodobacteraceae had the highest levels at the SI stage and then decreased gradually along with development. *Limnohabitans* has been reported to be present at a relatively high abundance in the epithelium of free-living *Hydra* and the gut microflora of *Daphnia magna* (Fraune and Bosch, 2007; Freese and Schink, 2011; Peerakietkhajorn et al., 2015). *Limnohabitans* has a prominent role in freshwater bacterioplankton communities due to their growth on alga-derived substrates as well as the high rates of substrate uptake and growth (Kasalický et al., 2013; Šimek et al., 2013). Its relative abundance was the highest during the SI stage, and it may be carried into the intestine and colonize with the plankton ingested by *P. clarkii*. *Brevundimonas* can promote the growth of algae like *Chlorella ellipsoidea* (Kim et al., 2018); in addition, some strains isolated from surface soil have been shown to produce hydroxylated astaxanthin, which is important for the growth and immunity of crayfish (Tao et al., 2006). Astaxanthin has been reported to rise rapidly during the SI stage of *Astacus leptodactylus* Eschscholtz to meet the needs of shrimp in the larval stage (Berticat et al., 2000). Furthermore, the high relative abundance of *Brevundimonas* in the SI stage corresponded to the high demand for astaxanthin in the larval stage.

An unclassified genus of Muribaculaceae was at a high level only at the TI stage, but it was present at low levels during the other stages (*p* < 0.05). As reported previously, Muribaculaceae play a variety of very important roles in the degradation of complex carbohydrates with the presence of a substantial and versatile set of carbohydrate-active enzymes in the genomes analyzed (Ormerod et al., 2016; Lagkouvardos et al., 2019). Moreover, the high abundance of Muribaculaceae may have an important metabolic potential during the TI period of frequent molting and rapid growth of crayfish. Therefore, further research is required.

*Thiothrix* was at a low level at the larval and adult stages but at a high level at the juvenile stage (*p* < 0.05). According to Temara et al. (1993) and Brigmon and Ridder (1998), *Thiothrix*-like bacteria presumably reduce the toxic effects of H_2_S produced in the preceding mainly anoxic loop of the intestine by re-oxidation (Temara et al., 1993; Brigmon and Ridder, 1998; Thorsen et al., 2003). However, in a study of intestinal microbiota in *Panulirus ornatus* larvae and juveniles, the multiplication of *Thiothrix* potentially hinders the ability of the animals to molt; combined with the added stress of the molt process, it likely results in reduced immune function, allowing opportunistic pathogenic *Vibrio* sp. to cause larval mortality (Payne et al., 2007), which also has been found in other marine invertebrates (Brigmon and Ridder, 1998). The peak period of morbidity and death in cultured *P. clarkii* is around May each year and is called the “Black May” disease; this disease presents with decreased crayfish activity and feeding, weakness, failure to molt, fluid build-up in the carapace and jejunum, and other features (Huang et al., 2020). Whether the high abundance of *Thiothrix* during the juvenile period is related to the high mortality rate during molting needs further investigation.

*Vogesella* increased gradually from the FI stage to the juvenile stage, reaching the highest level at the juvenile stage but sharply decreasing at the adult stage (*p* < 0.05). It is mainly present in an aqueous environment, has denitrifying properties (Bellini et al., 2018), and was previously found in the gut of snails (Osório et al., 2020).

*Dysgonomonas* and *Citrobacter* both had low levels at the first four stages, but they sharply increased at the adult stage (*p* < 0.05). *Dysgonomonas* is one of the intestinal symbionts present in animals such as *Lateolabrax japonicus*, *Kyphosus cinerascens*, and *Formosan subterranean termite* and is involved in the breakdown of the algae and vegetal material of the feed ingested by the host. *Dysgonomonas* accounts for more than 60% of the intestinal microbiota and may play an important role in the digestion of lignocellulose and ameliorate the gut environment for these animals (Husseneder et al., 2009; Han et al., 2019; Sun et al., 2019). In addition, *Dysgonomonas* is fastidious and grows very slowly, mainly as satellite bacteria, supporting that this dominant genus could be passed unnoticed unless a molecular approach to determine the microbial community composition is performed (Carda-Diéguez et al., 2014). Moreover, *Dysgonomonas* has been found to be dominant in crustaceans such as *P. clarkii* and *Eriocheir sinensis* as well as fly larvae (Feng et al., 2019; Gold et al., 2020). For *E. sinensis*, the relative abundance of *Dysgonomonas* was the highest in juveniles and relatively low in larvae and adults (Wang et al., 2018). *P. clarkii* is omnivorous, feeding mainly on zooplankton during the juvenile period and on large aquatic plants during the adult period (Li, 2006), which may be related to the large increase of *Dysgonomonas* at the adult stage. *Citrobacter* is a typical conditional pathogen widely found in nature, and its infection in *P. clarkii* activates the natural immune response of *P. clarkii* (Liu et al., 2020); however, in general, *Citrobacter* is normally dominant in the intestine of crayfish (Wu et al., 2021).

The more advanced and fully developed the animals are, the less of an impact the intestinal microbiota will have on them. The genetic specificity and diet of the host are the main factors that influence the mammalian intestinal microbiota, whereas for crustaceans, such as *E. sinensis*, *L. vannamei*, *P. clarkii*, *etc*., their intestinal microbiota is more susceptible to the environment (Stephens et al., 2015; Zheng et al., 2017; Chen et al., 2018; Wang et al., 2018; Shui et al., 2020; Tasnim et al., 2021). Furthermore, due to the feeding characteristics and shorter gut of crayfish, the intestinal microbiota of *P. clarkii* may be more closely related to the environment compared to those of fish and mammals. Therefore, compared to the phylogenetic investigation of communities by reconstruction of unobserved states, which focuses on metabolic functions, it may be more accurate to predict the functions of the intestinal microbiota of *P. clarkii* with FAPROTAX, which emphasizes biogeochemical processes. In this study, the most dominant functions of the intestinal microbiota in the three larval stages of crayfish were mainly chemoheterotrophy, aerobic chemoheterotrophy, and fermentation, with a relative abundance of more than 60%; meanwhile, the relative abundances of other functions were all less than 5%. The dominant functions in the juvenile stage included dark oxidation of sulfur compounds, dark sulfide oxidation, chemoheterotrophy, aerobic chemoheterotrophy, *etc*. The dominant functions in the adult stage included chemoheterotrophy, fermentation, aerobic chemoheterotrophy, animal parasites or symbionts, *etc*. In functional clustering, the three larval stages were clustered together, while the juvenile and adult crayfish were clustered separately according to the growth stage, indicating that the gut flora functions were more similar in the early development stages and changed as the host grew and developed. The functions of chemoheterotrophy, aerobic chemoheterotrophy, and fermentation were relatively advanced throughout growth and were conserved for the intestinal microbiota of *P. clarkii*. Different functions had distinct abundance patterns among the various growth stages – for example, the relative abundances of dark oxidation of sulfur compounds and dark sulfide oxidation were high at the juvenile stage but low in the other stages. Therefore, the relationship between the flora functions and the growth stage of the host needs to be further analyzed in conjunction with multi-omics.

As reported, the intestine of *P. clarkii* has a circular outline in transverse sections, six longitudinal ridges project inward from the wall of the organ, and there are many small, raised folds on the surface of the ridges, giving the lumen a stellate appearance. Each ridge consists of an adluminal epithelial layer and subepithelial connective tissue with glands. The remainder of the intestinal wall is configured of inner longitudinal and outer circular layers of striated muscle, which are embedded in a connective tissue that also forms the external boundary of the viscus (To et al., 2004). It has been reported that an increase in microbiota diversity may reflect the gut and immune maturation of the host (Yatsunenko et al., 2012). The microbiota is believed to be required for normal development; however, animals can develop without microbiota as shown by the existence of germ-free mice, rats, chickens, and pigs, but they have abnormal phenotypes (Dominguez-Bello et al., 2019). In this study, the intestinal tissue section analysis showed a not fully developed digestive system at the early stages. As the crayfish grew, the intestine developed, and the intestinal microbiota changed. Nevertheless, there was no sufficient evidence to determine the effect of the intestinal microbiota on intestinal development in *P. clarkii*. Therefore, further studies on sterile crayfish are needed.

## Conclusion

In the early stages of larval development of *P. clarkii*, the intestinal flora were similar. As the host grew, the intestine developed, the composition and function of the intestinal microbiota also changed, and the intestinal microbiota showed a consistent progression pattern in the larval and juvenile stages. Some microbiota showed stage specificity. However, further research is needed to increase the production and quality of crayfish for human consumption.

## Data Availability Statement

The datasets presented in this study can be found in online repositories. The names of the repository/repositories and accession number(s) can be found below: https://www.ncbi.nlm.nih.gov/bioproject/PRJNA723682.

## Author Contributions

MX contributed to writing and experiment. XC, ZW, and JY contributed to conduct the research. SZ and LX revised the manuscript. All authors contributed to the article and approved the submitted version.

## Conflict of Interest

The authors declare that the research was conducted in the absence of any commercial or financial relationships that could be construed as a potential conflict of interest.

## Publisher’s Note

All claims expressed in this article are solely those of the authors and do not necessarily represent those of their affiliated organizations, or those of the publisher, the editors and the reviewers. Any product that may be evaluated in this article, or claim that may be made by its manufacturer, is not guaranteed or endorsed by the publisher.
