# Supplementary figures and images for: Comparison of the Intestinal Microbiota During the Different Growth Stages of Red Swamp Crayfish (Procambarus clarkii)
Source: Front Microbiol. 2021 Sep 13;12:696281. doi: 10.3389/fmicb.2021.696281 (PMC8473915; doi:10.3389/fmicb.2021.696281)

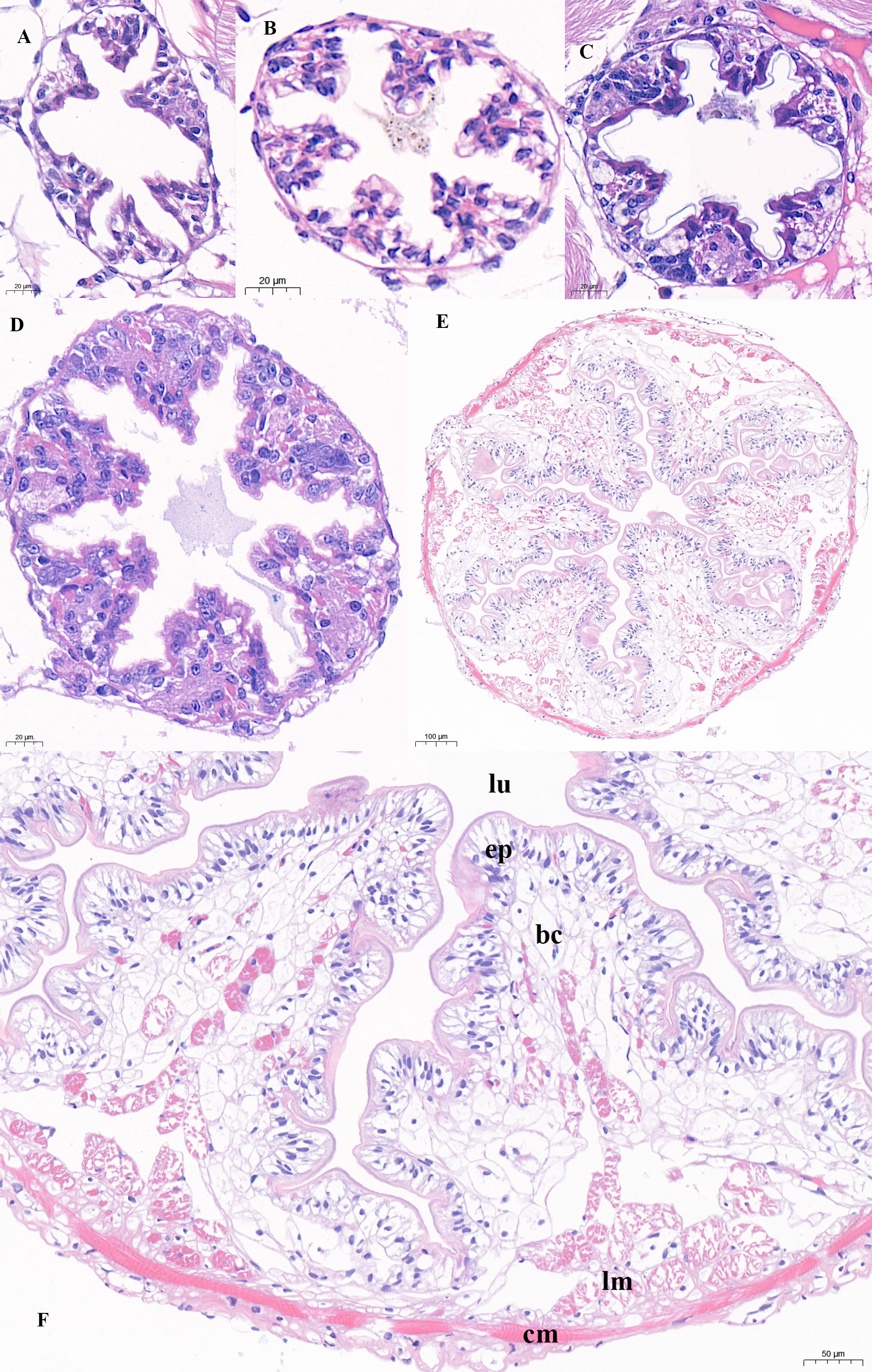

Supplement: Supplementary file 1 [file Image_1.jpg]
